# Report on palliative sedation medication usage: a survey of palliative care experts in Eight European countries

**DOI:** 10.1186/s12904-024-01484-6

**Published:** 2024-06-20

**Authors:** Éva Pozsgai, Eduardo Garralda, Csilla Busa, Sheila Payne, Jeroen Hasselaar, Daniela Mosoiu, Séverine M. Surges, Michaël Van der Elst, Sebastiano Mercadante, Carlos Centeno, Ágnes Csikós

**Affiliations:** 1 Department of Primary Health Care, University of Pecs Medical School, Pécs, Hungary; 2 Department of Public Health Medicine, University of Pécs Medical School, Pecs, Hungary; 3 ATLANTES Global Observatory of Palliative Care, Institute for Culture and Society, University of Navarra, Pamplona, Spain; 4 IdiSNA, Instituto de Investigación Sanitaria de Navarra, Pamplona, Spain; 5 Institute of Primary Health Care, University of Pécs Medical School, Pécs, Hungary; 6 International Observatory On End of Life Care, Lancaster University, Lancaster, UK; 7 Department of Primary Healthcare, Radboud University Medical Center, Nijmegen, The Netherlands; 8 Transylvania University Brasov, Hospice Casa Sperantei, Brasov, Romania; 9 Department of Palliative Medicine, University Hospital Bonn, Bonn, Germany; 10 Department of Oncology, Laboratory of Experimental Radiotherapy, KU Leuven, Leuven, Belgium; 11 Main Regional Center for Palliative Care, La Maddalena Cancer Center, Palermo, Italy

**Keywords:** Palliative sedation, Medication, Medicine, Palliative care, Opioid

## Abstract

**Background:**

The practice of palliative sedation continues to raise ethical questions among people, which in turn leads to its varied acceptance and practice across regions. As part of the Palliative Sedation European Union (EU) project, the aim of the present study was to determine the perceptions of palliative care experts regarding the practice of palliative sedation in eight European countries (The Netherlands, Belgium, Germany, UK, Italy, Spain, Hungary, and Romania).

**Methods:**

A specifically designed survey, including questions on the most frequently used medications for palliative sedation, their availability per countries and settings, and the barriers and facilitators to the appropriate practice of palliative sedation was sent to expert clinicians involved and knowledgeable in palliative care in the indicated countries. A purposive sampling strategy was used to select at least 18 participating clinicians per consortium country. Descriptive statistical analysis was conducted on the survey data.

**Results:**

Of the 208 expert clinicians invited to participate, 124 participants completed the survey. Midazolam was perceived to be the most frequently used benzodiazepine in all eight countries. 86% and 89% of expert clinicians in Germany and Italy, respectively, perceived midazolam was used “*almost always”*, while in Hungary and Romania only about 50% or less of the respondents perceived this. Levomepromazine was the neuroleptic most frequently perceived to be used for palliative sedation in the Netherlands, Spain, Germany, and the United Kingdom. Between 38- 86% of all eight countries´ expert clinicians believed that opioid medications were “*almost always”* used during palliative sedation. The perceived use of IV hydration and artificial nutrition “*almost always*” was generally low, while the country where *both* IV hydration and artificial nutrition were considered to be “*very often*” given by a third of the expert clinicians, was in Hungary, with 36% and 27%, respectively.

**Conclusions:**

Our study provides insight about the differences in the perceived practice of medication during palliative sedation between eight European countries. In countries where palliative care services have been established longer perceptions regarding medication use during palliative sedation were more in line with the recommended European guidelines than in Central and Eastern European countries like Romania and Hungary.

**Supplementary Information:**

The online version contains supplementary material available at 10.1186/s12904-024-01484-6.

## Background

Palliative sedation is defined as a treatment with sedative medications used to alleviate suffering from refractory symptoms of a patient with a terminal illness by reducing their consciousness [1]. The components of palliative sedation are determined by the depth (light or deep) and length (intermittent or continuous) of sedation [2], all individually tailored to the patient based on the characteristics of the patient’s symptoms, the assessment of the multidisciplinary care team and the patient’s and family’s wishes [2]. Medications used during palliative sedation include benzodiazepines, neuroleptics, and -when required for certain symptoms—co-medications, such as opioid and non-opioid medications [3]. Intravenous hydration, artificial nutrition, and additional medications may complement palliative sedation therapy [4].

Due to a lack of consensus for the standardized use of palliative sedation, the European Association for Palliative Care (EAPC) recommended a 10-point framework to facilitate the development of high-quality local procedural guidelines in 2009 [5], which was subsequently followed by the development of European guidelines, mostly based on expert consensus [6]. Yet, despite its well-defined differentiation from euthanasia, the practice of palliative sedation continues to raise ethical questions among people from different cultural and religious backgrounds, which in turn leads to its varied acceptance and practice across regions [7–9]. Although 10–18% of deaths in Europe are estimated to have been associated with the use of palliative sedation [10, 11], data on their exact numbers, their use across countries, and the factors that are promoters or obstacles to its use are lacking.

The Palliative Sedation European Union (EU) project (https://palliativesedation.eu) was initiated with the participation of researchers from eight European countries to investigate various aspects of palliative sedation.

As part of this project, the aims of the present study were to determine the most frequently used medications for palliative sedation, their availability per countries and settings, and the barriers and facilitators to the appropriate practice of palliative sedation as perceived by palliative care experts, in eight European countries.

## Methods

### Study design

The study was designed as part of the 5-year Palliative Sedation project, which includes researchers from eight participating European countries (Belgium, Germany, Hungary, Italy, the Netherlands, Romania, Spain, and the United Kingdom) investigating the practice of palliative sedation (https://palliativesedation.eu). The study received ethical approval prior to its initiation and has received funding from the European Union’s Horizon 2020 research and innovation program under grant agreement No. 825700. The present study focused on investigating expert’s perceptions of the use of main medications for palliative sedation. A specifically designed survey was sent to palliative care expert clinicians (physicians and nurses) from all eight participating European countries.

### Survey

In preparation for the survey, an online search was conducted for previously published reports on national and/or international questionnaires regarding the practice of palliative sedation. The search identified articles published since 2005 on PUBMED and Google Scholar using the following search terms: [“palliative sedation” AND “survey”] [12]. The search identified 19 studies, from which two -including the guidelines by the Spanish Collegiate Medical Organization and the Spanish Society for Palliative Care and the EAPC framework for palliative sedation- that inspired the questions on medications used in palliative sedation. Thus, the list of medications and co-medications suggested for the survey were based on the report by the Spanish Collegiate Medical Organization and the Spanish Society for Palliative Care [13] and the EAPC framework for palliative sedation [5]. Following the online search, an initial draft of questions, along with suggestions for further items, was reviewed by the palliative care experts. The questions relating to palliative sedation included both continuous and intermittent (as well as light and deep) sedation. Questions regarding the barriers and facilitators to the use of the appropriate medication for palliative sedation were added based on the experience of the ATLANTES Global Observatory of Palliative Care. All survey items were discussed and reviewed by the consortium partners during the consortium meeting held in March 2019. The protocol of the study as well as the survey were circulated and revised by consortium members in the Fall of 2019, to ultimately obtain the finalized versions of the study protocol and questionnaire. The finalized list of altogether 36 questions of the survey, including the list of four questions pertaining to the present study (as shown in Supplementary file 1), regarding medication use in palliative sedation was approved by all project researchers. The survey was written in English and designed in Survey Monkey and included the following questions:

1. Please, indicate the frequency of use and availability per setting* of parenteral formulation of the following medications in your country (midazolam, lorazepam, flunitrazepam, other benzodiazepines*; chlorpromazine, levomepromazine, other neuroleptics/antipsychotics*; anticonvulsants, fenobarbital, pentobarbital, other barbiturates; propofol, other anaesthesics*, antihistamines, other anticholinergics.
  “Home settings” were defined as all types of care provided for the patient in the patient’s home, and “Hospital settings” meant all types of inpatient care (including inpatient hospice care) provided for the patient.
2. *Which of the following co-medications and treatments are used with palliative sedation*? (Opioid medication, Non-opioid medication, intravenous (IV) hydration, Artificial nutrition, Antibiotic treatment, Antithrombotic medications).
  In these two topics, response options included the following frequency categories: “Almost never, rarely, sometimes, often, or nearly always” and from the following settings: “home, hospital or both”.
3. *Which is the barrier that hinders palliative sedation to be carried out with the right medication?* (main barrier and other barriers).
4. *Which is the facilitator that allows palliative sedation to be carried out with the right medication?* (main facilitator, other facilitators).

The detailed survey questions are shown inSupplementary file 1.

### Selection of survey respondents

A purposive sampling strategy was used to select participants for the survey. Expert clinicians were identified with the listed disciplinary profiles, based on the researchers’ consensus: clinicians working at a nursing home, hospice, hospital, or home care settings and working in one of the following areas of medicine: anesthesiology, intensive care, internal medicine, oncology, palliative medicine, and primary care. Our goal ideally was to choose at least one physician and one nurse from each indicated field of medicine (Table 1). Potential participants for the study were identified by the research team from each country using the same matrix, as shown below, and described previously [14].

**Table 1:** Participating expert clinicians’ matrix showing the disciplinary profiles (field of medicine, setting of work, and profession)

Field	Setting	Profession
Anesthesiology	Hospital	Physician
Anesthesiology	Hospital	Nurse
Intensive care	Hospital	Physician
Intensive care	Hospital	Nurse
Internal Medicine	Hospital	Physician
Internal Medicine	Hospital	Nurse
Oncology	Hospital	Physician
Oncology	Hospital	Nurse
Primary care	Primary	Physician
Primary care	Primary	Nurse
Palliative care	Hospital	Physician
Palliative care	Hospital	Nurse
Palliative care	Home	Physician
Palliative care	Home	Nurse
Palliative care	Nursing home	Physician
Palliative care	Nursing home	Nurse
Palliative care	Hospice	Physician
Palliative care	Hospice	Nurse

The recommended sources for identifying candidates were the following: 1) The National Palliative Care Association and the Nursing Palliative Care Association, 2) Members of workforces on palliative sedation, guideline authors, or authors of related articles, and 3) Members of National Anaesthesiology, Intensive Care, Primary Care, Oncology, and Internal Medicine Societies [14]. Since 18 different disciplinary profiles were determined, at least 18 clinicians per consortium country working in palliative care and knowledgeable regarding the most frequently used medications on palliative sedation were identified by the researchers in each consortium country as candidates for the survey.

Subsequently, the identified expert clinicians were invited by letter to take part in the study, in which a letter of introduction, consent form and invitation to participate in the survey were sent in December 2019. If they responded and accepted, the survey link was sent to the expert clinicians on January 30th, 2020, with a one month-period deadline. One letter of reminder was sent during February 2020 to those, who did not respond to the survey sent in January. Data collection was finished by March 15th, 2020.

Due to differences in the settings where palliative sedation is delivered between the countries, it was not possible to find enough representatives in all initially established categories. Since the aim of the study was to analyze the most frequently used medications in palliative sedation in each country, instead of focusing on specific fields of medicine, in such cases, the investigators recruited more representatives in certain categories [14].

### Survey respondents’ characteristics

There was a 60% response (*n* = 124) rate of the 208 expert clinicians invited to participate in the survey. Differences could be observed between the countries regarding the number of participants and their disciplinary profiles, due to dissimilarities in the practice of palliative care provision between the countries. (This phenomenon explains the differences in the missing profiles between the countries.) There was a mean of 19 participants per country, with the minimum and maximum number of participants between seven and 36. The detailed profiles of the expert clinicians participating in the study are shown in Table 2*,* as described in a previous publication [14].

**Table 2:** Disciplinary characteristics of the expert clinicians participating in the study [14]

Field	Setting	*Profession*	Countries^(a)^							
			BE	DE	ES	UK	HU	IT	NL	RO
**Anaesthesiology**	Hospital	*Physician*	1	1	1	-	1	3	1	1
		*Nurse*	-	-	-	-	-	-	-	-
**Intensive care**	Hospital	*Physician*	-	-	1	-	2	1	-	1
		*Nurse*	-	-	1	-	-	1	-	-
**Internal Medicine**	Hospital	*Physician*	-	-	1	-	-	1	-	-
		*Nurse*	-	-	1	-	1	1	1	-
**Oncology**	Hospital	*Physician*	-	-	1	-	3	2	1	3
		*Nurse*	-	-	1	-	1	1	-	-
**Primary care**	Primary	*Physician*	1	1	2	-	1	1	1	1
		*Nurse*	1	-	1	-	-	-	-	-
**Palliative care**	Hospital	*Physician*	1	1	1	2	4	6	2	4
		*Nurse*	2	-	1	-	1	-	6	1
	Home care	*Physician*	1	1	1	2	1	5	-	2
		*Nurse*	1	1	1	-	1	1	2	1
	Nursing home	*Physician*	1	1	1	-	-	-	2	-
		*Nurse*	-	-	1	-	-	-	-	-
	Hospice	*Physician*	1	1	1	9	1	13	3	1
		*Nurse*	-	-	1	-	-	1	-	1
**Number of experts per country**^**(b)**^	-	-	**10**	**7**	**18**	**9**	**11**	**36**	**16**	**17**

^a^BE Belgium; DE Germany; ES Spain; UK United Kingdom; HU Hungary; IT Italy; NL Netherlands; RO Romania

^b^Some of the participating experts worked in two different settings, therefore, the data from diverse countries does not necessarily match the number of experts per country

### Data analysis

#### Use of medications and co-medications and availability per setting (topics 1 and 2)

Descriptive statistical analysis was conducted on the survey data. Percentages of reported frequency of use of medications in all categories (almost always, very often, sometimes, rarely, almost never, not available (n/a) were calculated per country and per medication and per co-medication and per treatment. All response options were counted for frequency per country (*n* = number of respondents reporting each frequency category). Percentages were then calculated by dividing the number of respondents reporting a frequency, by the total number of respondents of the country, which was the only appropriate method to compare countries given that each country had different number of respondents. The same process was followed to estimate each medication´s and co-medication’s availability per setting. To facilitate interpretation “Very often” and “Always” categories were collapsed into one category: “Almost always” for the purpose of the study, and the figures show only high frequencies as the aim of the study was to identify most frequently used medications, not uncommon ones.

#### Barriers and facilitators to the use of the appropriate medications in palliative sedation (topics 3 and 4)

Barriers and facilitators were analyzed using framework content analysis. Codenames were agreed on, and a frequency description presented for each code. All reported statements were coded by two independent researchers blinded to each other’s assessment. Discrepancies in coding were solved by consensus between the two researchers.

## Results

### Perceived use of benzodiazepines in palliative sedation

**Fig. 1:**
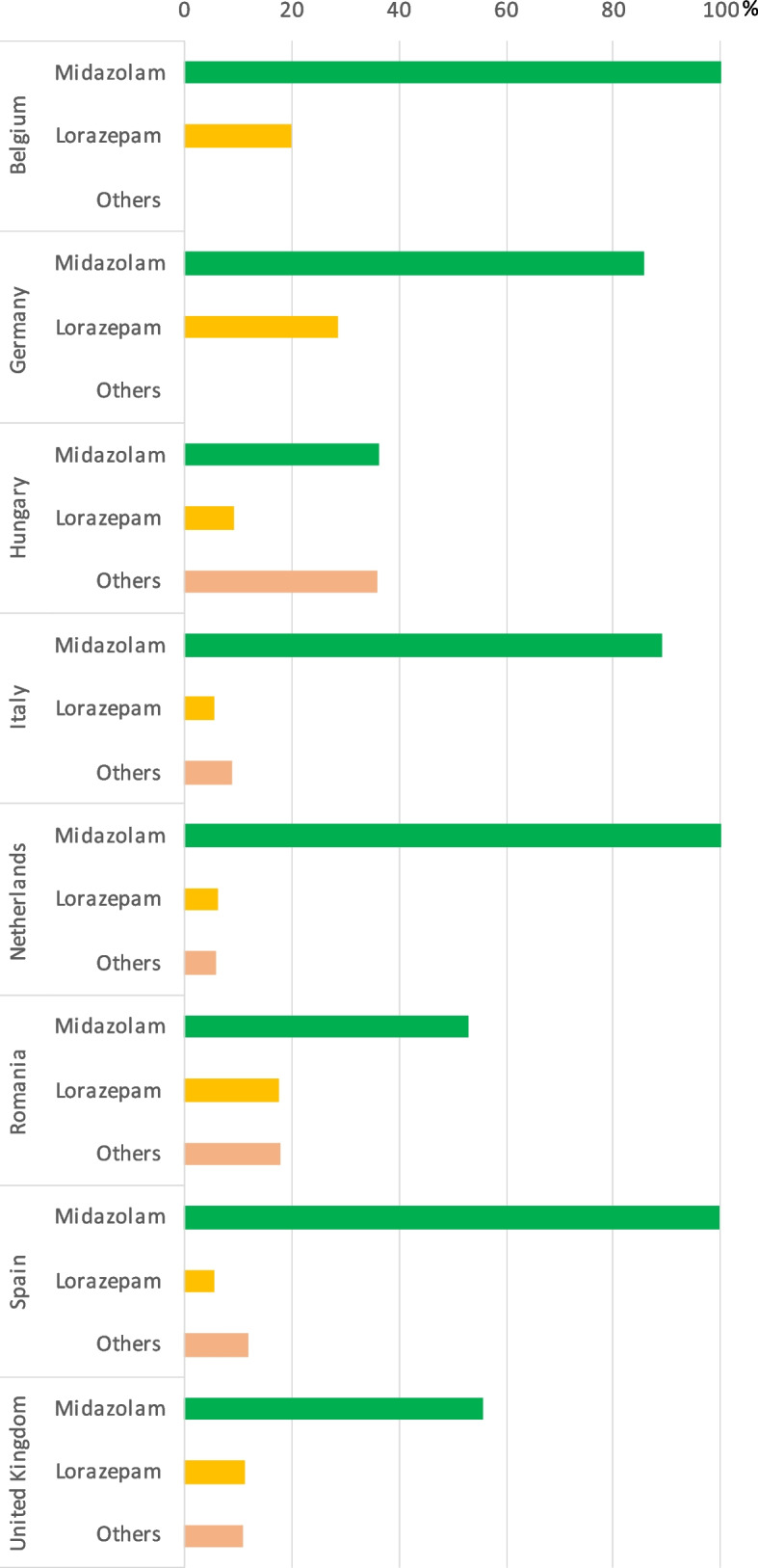
Percentage of the countries’ expert clinicians reporting perceived use of benzodiazepines “almost always”. No bars indicate that none (0%) of the expert clinicians perceived the given medication as being used “almost always”

Midazolam was perceived to be the most frequently used benzodiazepine in all eight countries. In the Netherlands, Belgium, and Spain all expert clinicians reported that midazolam was used *almost always* by clinical staff when performing palliative sedation. 86%-89% of expert clinicians in Germany and Italy, respectively, perceived midazolam was used *almost always*, while in Hungary and Romania only about 50% or less of the respondents thought that midazolam was used almost always. The second most frequently used benzodiazepine was lorazepam in many countries; however, it was considered to be used *almost always* by less than 30% of the expert clinicians across countries. Other benzodiazepines were much less frequently perceived to be used than the first two, except in Hungary. (Fig. 1).

**Fig. 2:**
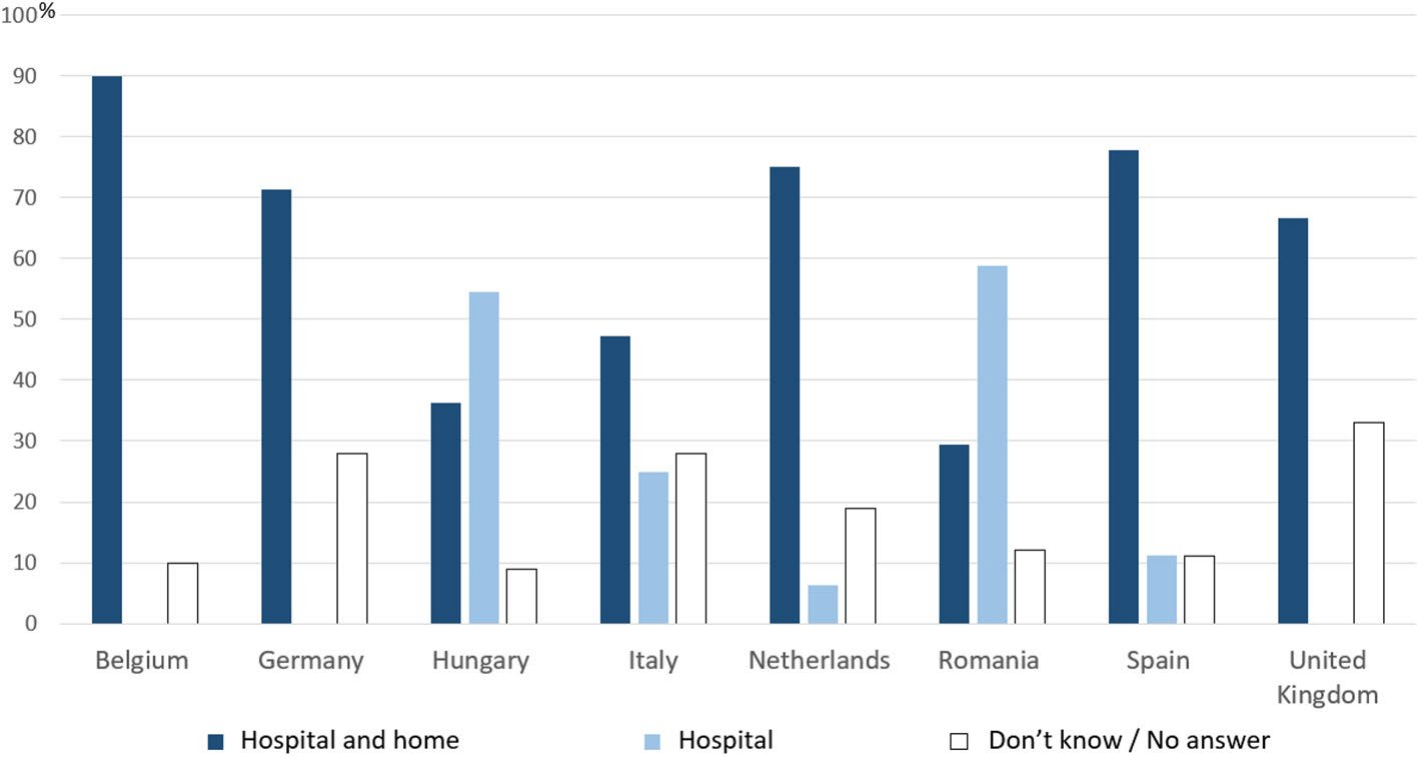
Perceived availability of midazolam in different settings by expert clinicians. Light blue bars indicate the perceived percentage of midazolam availability at just the hospital, dark blue bars indicate the perceived availability of midazolam at both home and hospital settings. White bars show the percentage of expert clinicians who did not give an answer or indicated “don’t know” as an answer

When analyzing the expert opinion about the availability of the most frequently used benzodiazepine, midazolam, in different settings, we found that in the majority (5/8) of the countries, midazolam was perceived to be available in both hospital and home settings by more than 67% of the expert clinicians, with midazolam’s availability in both settings perceived to be as high as 90% in Belgium. The countries where both the perceived hospital and home availability was lowest, were Italy, Hungary, and Romania. Interestingly, while the perceived availability of midazolam in both settings was lowest in Romania and Hungary (below 40% in both cases), in contrast its perceived exclusive availability in hospital settings was highest in these countries: 55% in Hungary, 59% in Romania (Fig. 2).

### Perceived use of neuroleptics in palliative sedation

**Fig. 3:**
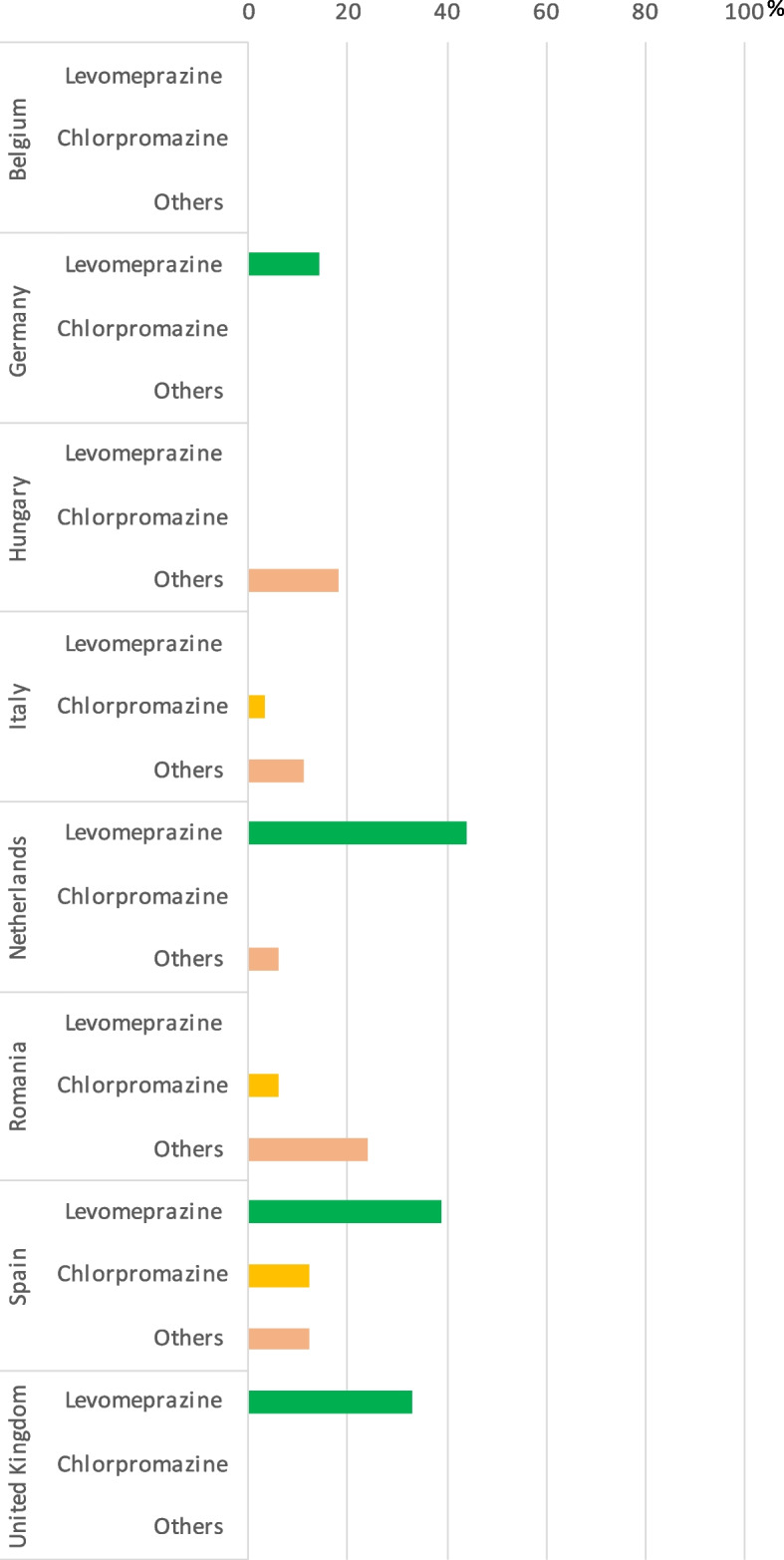
Percentage of the countries’ expert clinicians reporting perceived use of neuroleptics “almost always”.No bars indicate that none (0%) of the expert clinicians perceived the given medication as being used “almost always”

The analysis of neuroleptics showed that there were great variations in their perceived use between the countries. Levomepromazine was the neuroleptic most frequently perceived to be used for palliative sedation in half of the countries: in the Netherlands, Spain, Germany, and the United Kingdom. Yet, even in these countries, it was thought to be used *“almost always”* by less than 44% of the expert clinicians. Chlorpromazine and other neuroleptics were believed to be used “*almost always*” by less than a quarter of the respondents in all countries. At least one of the investigated neuroleptics (levomepromazine, chlorpromazine or members of the “others” group), was perceived to be used less frequently than *“almost always*” in all countries, with all expert clinicians in Belgium perceiving the use of all neuroleptics less frequent than “*almost always*”. (Fig. 3).

**Fig. 4:**
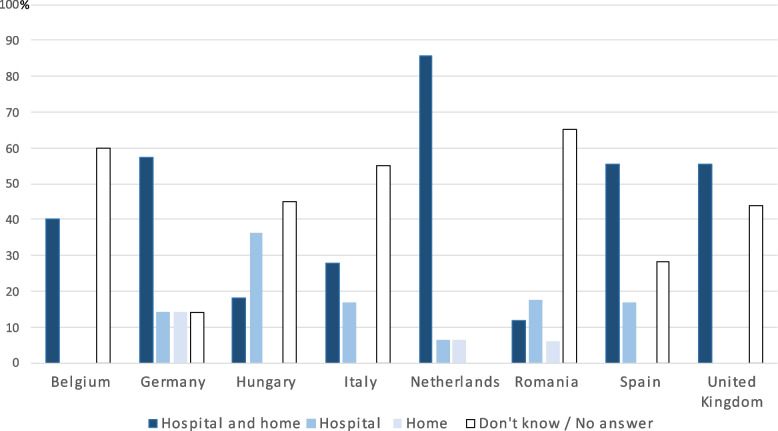
Perceived availability of levomepromazine in different settings by expert clinicians. Light blue bars indicate the perceived percentage of levomepromazine availability at just the hospital, dark blue bars indicate the perceived availability of levomepromazine at both home and hospital settings. White bars show the percentage of expert clinicians who did not give an answer or indicated “don’t know” as an answer

Regarding the perceived availability of the most frequently used neuroleptic, levomepromazine, per setting, this neuroleptic was thought to be available in both hospital and home settings in the countries where it was also perceived as the most frequently, “almost always” used neuroleptic; in the Netherlands, Germany, Spain and the United Kingdom. There was a fairly large proportion of expert clinicians who did not answer the question related to availability or who replied “don’t know” to the question, and these percentages were highest in the countries where it was perceived to be used infrequently: in Romania, Belgium, Italy, and Hungary. (Fig. 4).

### Perceived use of co-medications and additional treatments (IV hydration, artificial nutrition) in palliative sedation

Next to sedatives, an array of co-medications and treatments may be used during palliative sedation, from which pain medication, antibiotic- and antithrombotic medications along with hydration and nutrition therapies are the most important.

Between 38- 86% of all eight countries´ expert clinicians believe that opioid medications are “*almost always”* used during palliative sedation, with the highest percentages above 84% found in Germany, Italy, and Spain and the lowest in the Netherlands with 38%.

The co-medications perceived to be used second most frequently were non-opioid analgesics, with the highest percentages of expert clinicians believing its use during palliative sedation occurred “almost always” in Hungary (63%) and Germany (43%).

The perceived use of antibiotics during palliative sedation was low in all countries, with less than 11% believing it was used “almost always”.

Similarly, antithrombotic medication was perceived to be used infrequently in almost all countries (10% >), excepting Hungary, where more than a third of the expert clinicians (36%) perceived it was used “almost always”.

**Fig. 5:**
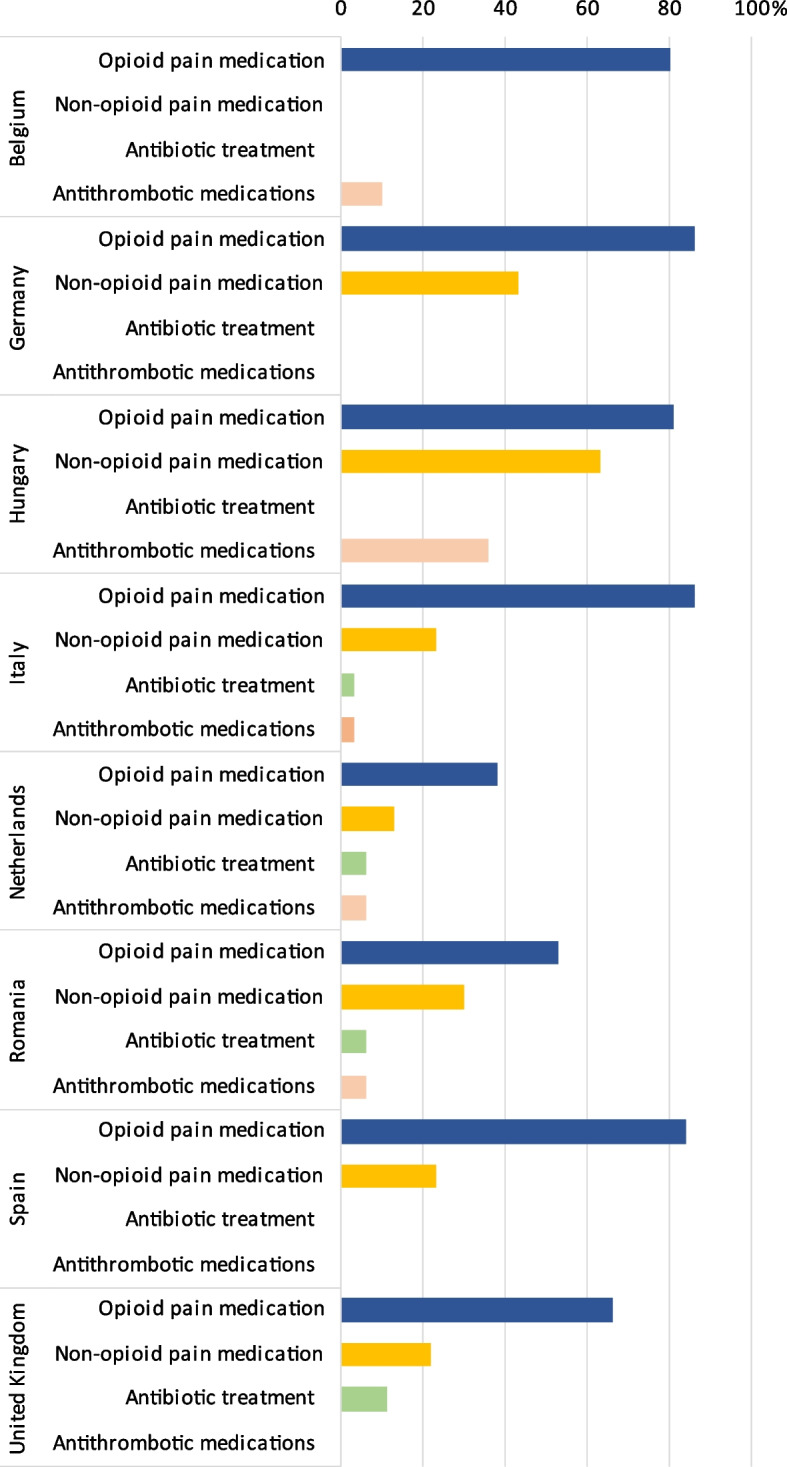
Percentage of the countries’ expert clinicians reporting perceived use of co-medications “almost always”. No bars indicate, that none (0%) of the expert clinicians perceived the given medication as being used “almost always”

Figure 5 shows the percentage of the countries’ expert clinicians reporting perceived use of co-medications.

**Fig. 6:**
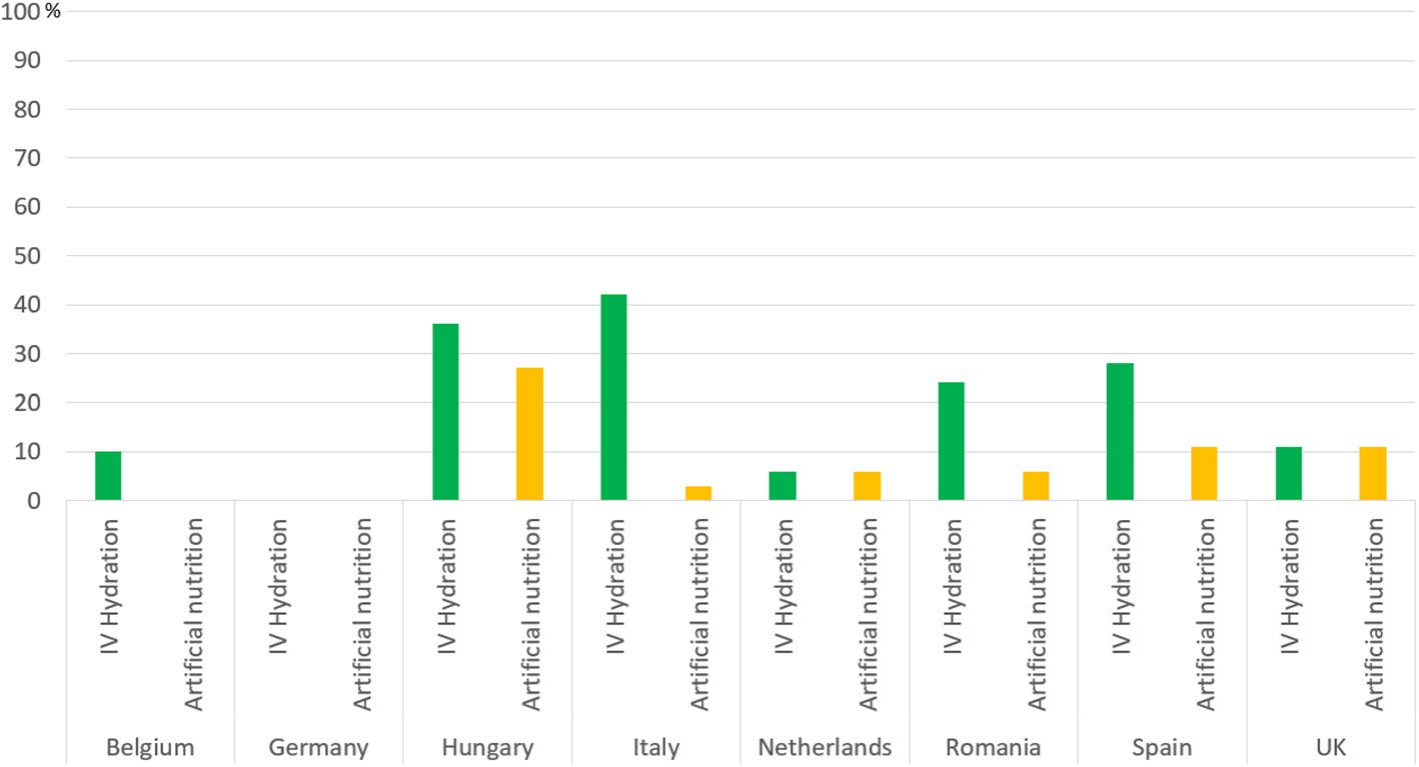
Percentage of the countries’ expert clinicians reporting perceived use of IV hydration and artificial nutrition “almost always”. No bars indicate that none (0%) of the expert clinicians perceived the given medication as being used “almost always”

The perceived use of IV hydration and artificial nutrition “*almost always*” was generally low, with their use being very low (below 10%) in four countries: Belgium, Germany, The Netherlands, and the United Kingdom. In general, IV hydration was perceived to be given more frequently than artificial nutrition. IV hydration was believed to be given “*almost always*” by the highest percentage of Italian expert clinicians (42%) compared to other countries. However, the country where *both* IV hydration and artificial nutrition were considered to be “*very often*” given by a third of the expert clinicians, was in Hungary, with 36% and 27%, respectively (Fig. 6).

### Barriers and facilitators to the correct use of medication in palliative sedation

When expert clinicians were asked to describe which, they thought were the main perceived barriers to the correct use of medication in palliative sedation, ‘lack of adequate knowledge’ was considered to be the main barrier in six out of the eight countries, with the experts in Romania mentioning limited ‘availability of certain medications’ and ‘no major barrier’ in Germany, as most important barriers to palliative sedation. Availability of certain medications, availability of PC specialists/teams, legal issues surrounding palliative sedation and cultural or religious issues were believed to be other main barriers to the correct use of medication in palliative sedation (Table 3).

**Table 3:** Main perceived barriers to the correct use of medication in palliative sedation

Countries	%^(a)^	Most frequently perceived barrier	%^(a)^	2nd most frequently perceived barrier	%^(a)^	3rd most frequently perceived barrier
Belgium (12)	42%	Lack of knowledge, competence^(b)^	17%	Availability of certain medicines^(c)^	8% 8% 8% 8%	Lack of experience Lack of education / training Lack of / unclear guidelines Legal issues
Germany (13)	38%	None	23%	Others	15%	Lack of experience
Hungary (12)	25%	Lack of knowledge, competence^(b)^	17% 17% 17%	Availability of certain medicines^(c)^ Availability of PC specialists/team^(d)^ None	8% 8%	Lack of experience Legal issues
Italy (35)	46%	Lack of knowledge, competence^(b)^	14% 14%	Legal issues Cultural or religious issues	9%	Availability of certain medicines^(c)^
Netherlands (21)	38%	Lack of knowledge, competence^(b)^	24%	Availability of certain medicines^(c)^	19%	Reimbursement of medicines
Romania (28)	32%	Availability of certain medicines^(c)^	18% 18%	Lack of knowledge, competence^(b)^ Lack of education / training	11% 11%	Legal issues Cultural or religious issues
Spain (25)	32%	Lack of knowledge, competence^(b)^	20%	Legal issues	16%	Availability of PC specialists/team^(d)^
United Kingdom (7)	29% 29%	Lack of knowledge, competence^(b)^ Cultural or religious issues	14% 14% 14%	Lack of education / training Lack of / unclear guidelines Fear of using medicines	-	-

*PC* Palliative care

^a^Percentage of responses for the given country

^b^Lack of (adequate) knowledge, competence

^c^Availability of (certain) medicines in certain settings in appropriate form

^d^Availability of palliative care specialists/team (for consultation / collaboration)

When analyzing the factors perceived to be the main facilitators to the correct use of medication in palliative sedation, we found that the most frequently reported facilitators were the education and training of professionals along with adequate knowledge (in Belgium, Germany, Romania, Hungary, the UK and Spain) and the availability of medications and specialized palliative care services. The existence and implementation of guidelines and protocols were also considered to be important facilitators, particularly in the Netherlands (Table 4).

**Table 4:** Main facilitators to the use of medications for palliative sedation across Europe

Countries	%^( a)^	Most frequently perceived facilitator	%^(a)^	2nd most frequently perceived facilitator	%^(a)^	3rd most frequently perceived facilitator
Belgium (12)	45%	Availability of spec. PC teams^(b)^	27%	Existence of guidelines	18%	Education and training
Germany (13)	33% 33%	Education and training Existence of guidelines	17% 17%	Trained PC staff Availability of medicines	-	-
Hungary (12)	25% 25% 25%	Knowledge Availability of medicines Education and training	13% 13%	Availability of spec. PC teams^(b)^ Existence of guidelines	-	-
Italy (35)	19%	Others	15%	Education and training	12% 12% 12%	Knowledge Availability of spec. PC teams^(b)^ Availability of medicines
Netherlands (21)	21%	Implementation of guidelines / protocols	14% 14% 14% 14% 14%	Knowledge Education and training Availability of medicines Existence of guidelines Reimbursement of medicines	-	-
Romania (28)	21% 21% 21%	Availability of medicines Education and training Trained PC staff	14%	Availability of spec. PC teams^(b)^	7% 7%	Knowledge Legal issues
Spain (25)	33%	Education and training	17% 17%	Availability of spec. PC teams^(b)^ Others	11%	Knowledge
United Kingdom (7)	20% 20% 20% 20% 20%	Knowledge Education and training Communication issues Reimbursement of medicines Others	-	-	-	-

*PC* Palliative care

^a^Percentage of responses for the given country

^b^Availability of specialized PC teams

Education and knowledge were perceived to be a facilitator by 50%-50% of the experts in the UK. The availability of palliative care teams, referring to the presence of specialized palliative care teams, was perceived to be a facilitator by as many as 45% of the Belgian experts. Finally, the availability of medications, representing the general availability of medications, was thought to facilitate palliative sedation mostly by experts in Romania (21%).

## Discussion

To our knowledge this is the first study to investigate and compare the perceptions of medication use and barriers and facilitators of appropriate medication use during palliative sedation, in eight European countries. We found distinct patterns of medication use, with perceptions of their use more in line with current EAPC recommendations [5] in the countries where palliative care services had been established longer (The Netherlands, Belgium, Germany, and the UK). Perceptions of the main barriers to the appropriate use of palliative sedation showed that lack of adequate knowledge and availability of certain medications in certain settings were considered as the main obstacles across all the studied countries, with the highest percentages of expert clinicians having this perception in Italy and Romania. It must be noted, however, that the observed differences in the patterns of medication use may have been influenced by differences between the practice of palliative and hospice care provision between the countries and the interpretation of the term ‘palliative sedation’, between the respondents from the different countries.

Palliative sedation can be indicated when patients with a terminal illness, experience refractory physical and/or psychological symptoms – and in some cases existential suffering- which cannot be adequately managed by other supportive and palliative treatment methods [15]. These symptoms include pain, dyspnea, delirium, agitation, anxiety and vomiting and palliative sedation may be indicated in 10–50% of the cases [16, 17]. According to the 2009 EAPC framework, benzodiazepines and, in cases of delirium, (additional) neuroleptics should be used primarily for achieving sedation [5], which is also recommended by guidelines in the Netherlands and Spain [18–20]. Among benzodiazepines, midazolam and among neuroleptics, levomepromazine are the medications primarily recommended [18–20]. Possible alternatives to midazolam are diazepam, lorazepam, clonazepam and flunitrazepam [15, 18–21]. Chlorpromazine, clotiapine, [22] and promethazine [21] are possible alternatives to levomepromazine, as described in the 2009 EAPC framework and the Italian, Spanish, Belgian and Dutch guidelines.

Our study showed that in line with the mentioned guidelines, midazolam was perceived to be used most frequently, i.e. “almost always”, in all eight countries, however, there were notable differences. The highest proportions with perceived midazolam use were mostly the Western European countries (The Netherlands, Belgium, and Germany) and South-European countries (Spain, Italy), while experts in the Central and Eastern European countries, like Romania and Hungary considered the primary use of midazolam much less frequent, with “almost always” use of midazolam being less than 40% in Hungary and similar to “other” less frequently used benzodiazepines. [23]. These perceived differences in the availability and use of midazolam may be explained by differences between the countries regarding the types of palliative and hospice care service provision in different settings as well as what medications can be prescribed and legally given at home [24–26]. In addition, the much more recent establishment of palliative care services – and concomitant guidelines- in the Central -European countries investigated in our study compared to countries where palliative care has been present for decades, like the Netherlands or the UK, could also have led to the observed differences [27]. According to a systematic review, availability of medications for palliative sedation at home are important, since it is both a feasible treatment option and an option for improving care for those who do not wish to be treated in a hospital setting [28].

In our analysis, the patterns in neuroleptic use were much less uniform among the countries, than for midazolam, since the primarily recommended levomepromazine [5, 18–20] was perceived to be used for palliative sedation “almost always” by less than half of the expert clinicians in the countries, the Netherlands, Spain, Germany, and the UK. The lower perception of the use of levomepromazine is supported by the EAPC recommendation, that midazolam is the first choice, when initiating palliative sedation and neuroleptics should mostly be used in cases of delirium or as a second line medication in combination with a benzodiazepine [5] Expert clinicians in Italy, Romania and Hungary did not report frequent perceived use of the first-line neuroleptic, levomepromazine, and mentioned “other medications” as being more frequently used. Knowledge, in general about the use of levomepromazine appeared to be lacking, which was demonstrated by the high proportion of key experts answering “don’t know” or leaving the question blank, when asked about levomepromazine’s perceived availability. The Hungarian guideline regarding palliative sedation does not specify the use of neuroleptics, which could explain the high number of “don’t know” answers [23].

Depending on the patient’s symptoms, co-medications may be administered during palliative sedation. Since pain and dyspnea are often cited as a refractory symptom, opioids may be given to complement treatment with sedatives [15]. However, the use of morphine should be used exclusively for the alleviation of pain and dyspnea and not as a sedative, as emphasized by the Dutch and Italian guidelines [18, 19, 21]. Although expert clinicians in most of the studied countries perceived the use of opioids to be relatively frequent during palliative sedation, the country, where expert clinicians perceived to be using them the most was in Hungary. In contrast, only 38% of the Dutch clinicians—in line with the mentioned guidelines- believed it to be used “almost always”. The observed difference in opioid use between the countries raises several questions: whether this was due to actual differences in the management of pain and dyspnea in terminally ill patients or to the inadequate assessment of pain by clinicians or that opioids – despite the national guidelines—were considered to be used instead of midazolam with the aim to sedate the patient.

Either way, our finding calls attention to the importance of the correct assessment of pain in terminally ill patients and to the importance of the use of opioid medications for the correct indications during palliative sedation.

According to the 2009 EAPC framework, “artificial hydration/nutrition therapy (….) should be individually decided through comprehensive evaluation of the patient’s wishes and the estimated benefits/harms” [5]. The ESMO clinical practice guidelines also stress the importance of decision making tailored to the individual patient’s needs, in consensus with the family members and the health care providers [15]. In contrast, the Belgian and Dutch guidelines state that administration of fluids is considered medically futile due to the terminal state of the patient’s illness when palliative sedation is deemed appropriate [15, 18, 22].

Reflecting the above-mentioned recommendations, both IV hydration and artificial nutrition were not perceived to be used frequently during palliative sedation in Belgium, the Netherlands, Germany, and the UK, in our study. South-European expert clinicians had a slightly different view: some frequency in IV hydration was perceived by clinicians in Spain, while clinicians in Italy reported the highest perceived use of IV hydration. The country where both IV hydration and artificial nutrition were considered to be used with notable frequency (about 30% “almost always”), was in Hungary. Although Hungarian palliative guidelines mirror the recommendations of the previously mentioned EAPC and ESMO [5, 15] guidelines, the perceived practice of hydration and nutrition in terminal patients appeared to show the opposite tendency. However, this phenomenon can partly be explained by specialized palliative care – and consequently palliative sedation- being available mostly in hospital settings, where the practice of artificial hydration and nutrition is more part of the routine practice in the care of patients than in the home setting. Similarly, the comparatively high perceived use of antibiotics in the UK could be due to the characteristics of the practice of palliative sedation; since palliative sedation is used intermittently to offer respite from suffering and not just near the end of life, this phenomenon may contribute to the higher perceived usage.

There is limited data regarding the factors that enable or impede the practice of palliative sedation. A study conducted in the UK investigated the ethical dilemmas hospice nurses faced during the administration of palliative sedation which concluded, that increasing the competence of nurses might facilitate the correct practice of palliative sedation [29]. Although palliative care services have been established longer in Western countries, like Belgium, lack of knowledge among GPs regarding correct palliative sedation practice and not being able to meet the needs of end-of-life care at home have been reported [30]. Corresponding with this study, we found, that lack of knowledge and unavailability of medications in certain settings were among the top four reasons perceived to be main barriers in the appropriate medication during palliative sedation—irrespective of the country- across all eight countries.

The perceived facilitators mirrored the perceived barriers to palliative sedation since improved education (to combat the barrier of lack of knowledge) and the availability of palliative care teams and availability of medications (to combat unavailability of medication and unavailability of a team specialized to perform palliative sedation) were perceived as the main facilitators of correct medication strategies during palliative sedation. The distribution of the top four reasons thought to be the main facilitators to the correct use of medication in palliative sedation showed that the education category reflects the need seen by experts to improve education on sedation medications at both the professional and the undergraduate levels.

These results therefore appear to emphasize the importance of education, possibly both at the graduate level at medical schools and at postgraduate levels as continuing medical education to educate clinicians on up-to-date evidence-based care regarding the medications-related guidelines for palliative sedation. Furthermore, studies on the practice of palliative sedation, like the present investigation, and subsequent measures aimed at informing those involved in health policy as well as the public. The results of these studies may also be important in improving the practice and acceptance of palliative sedation.

### Limitations

Our study has several limitations. The study was conducted on a comparatively small sample of clinicians and the number and profiles of respondents per country differed, nor were the number of clinicians representative of the countries’ population, which could all have led to bias. Furthermore, the choice of respondents (who were invited to participate) could have led to bias and the answers given by the respondents were not actual documentations of the practice of palliative sedation in the given country, rather a subjective opinion, of how the practice was perceived, potentially leading to memory bias. The majority of the respondents were based in inpatient care (specialist palliative care in hospitals or hospices) and were physicians, and this thus, was a source of bias, furthermore, the inclusion of nurses could also have introduced bias. The combination of the 'very often' and 'always' categories may have influenced results, by artificially increasing the frequency. Finally, differences in the interpretation of categories of frequencies (almost always, very often, sometimes, rarely, almost never, not available (n/a) and the term ‘palliative sedation’ between the respondents as well as differences in the structure of palliative and hospice care, the availability of palliative and hospice care in different settings and the actual practice of palliative and hospice care between the countries could also have influenced our results and led to bias.

Nevertheless, the aim of the study was to obtain the perceptions regarding medication use in palliative sedation, which could be collected from the survey.

## Conclusions

Our study provides information about the differences in the perceived practice of medication during palliative sedation. Generally, in countries where the palliative care services have been established longer, perceptions regarding the medication use during palliative sedation (use of sedatives, comedications and other treatments) were more in line with the recommended European guidelines (such as the EAPC framework) than in Central and Eastern European countries like Romania and Hungary. This, however, may also be because guidelines on palliative sedation were developed—and probably used in practice much longer in Western European countries. However, it must be noted, that the results of this study can only be interpreted within the context of its limitations.

Despite possibly more widespread perceived awareness of correct medication during palliative sedation, our study also demonstrated that even in Western European countries, further education regarding palliative sedation guidelines and awareness regarding them, need to be emphasized.

### Supplementary Information

Supplementary Material 1.

## Data Availability

The datasets generated and/or analyzed during the current study are not publicly due to data privacy of human patients but are available from the corresponding author on reasonable request.

## Abbreviations

BE: Belgium
DE: Germany
EAPC: European Association for Palliative Care
ES: Spain
EU: European Union
HU: Hungary
IT: Italy
IV: Intravenous
NL: Netherlands
RO: Romania
UK: United Kingdom
