# Supplementary material for: Report on palliative sedation medication usage: a survey of palliative care experts in Eight European countries
Source: BMC Palliat Care. 2024 Jun 20;23:154. doi: 10.1186/s12904-024-01484-6 (PMC11188245; doi:10.1186/s12904-024-01484-6)
Supplement: Supplementary file 1 — Supplementary Material 1. [file 12904_2024_1484_MOESM1_ESM.docx]

The survey

**The following questions are divided into sections according to the objectives of the study (existence**

**and use of national guidelines, regulations, and medications and equipment), plus an additional brief**

**section on the perception of barriers and opportunities to the use of Palliative Sedation guidelines.**

**The questions were carefully chosen following a review of previous national and international surveys**

**and questionnaires about Palliative Sedation. We selected questions in each section after review by**

**the research team and discussion with all project partners.**

**The survey consists of 36 questions. You can exit the survey at any time and your responses will be**

**saved once you have finished each section. Please answer the questions to the best of your ability.**

**Some of the questions may require you to give your best estimate as an expert in Palliative Sedation**

**issues in your country.**

**We have provided definitions before each section as well as additional information at the end of the**

**section in order to assist you. Please read the definitions before starting to answer the questions. If**

**you have any comments that you wish to add, there is a comment box at the end of the whole survey.**

**You can also download the questionnaire in a**

[**t**](https://drive.google.com/a/unav.es/file/d/1ReySW6OmdsXxQt5A88aHqGqp5cwC2Zp8/view?usp=sharing)

**pdf forma**

**, so that you can read the questions before**

**you complete it or by using the pdf form and returning it to us via e-mail if you prefer.**


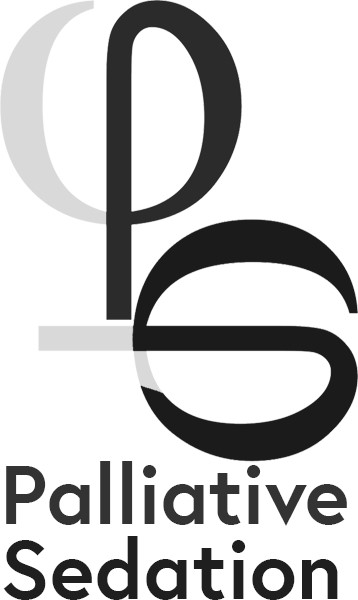


**Name (as you would like**

**it to appear in the**

**Acknowledgements)**

**Affiliation (as you would**

**like it to appear in the**

**Acknowledgements)**

**Country**

1

.

**Contact Information**

*

2

.

**You will not be formally acknowledged in the acknowledgement section of the final report. If you**

**wish to be publicly acknowledged please tick the box below.**

I would like to publicly acknowledged.


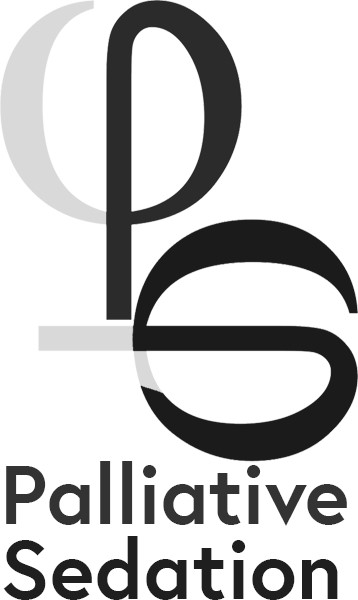


|  |
| --- |
| SECTION 3: Palliative Sedation Medication and Equipment |

# Definitions and relevant information

Medical equipment used for the specific purposes of diagnosis and treatment of disease or rehabilitation following disease or injury; it can be used either alone or in combination with any accessory, consumable or other piece of medical equipment. Medical equipment excludes implantable, disposable or single-use medical devices. (WHO, 2019). We are following the list of medications and comedications of OMC&Secpal 2011 and Cherny 2009.


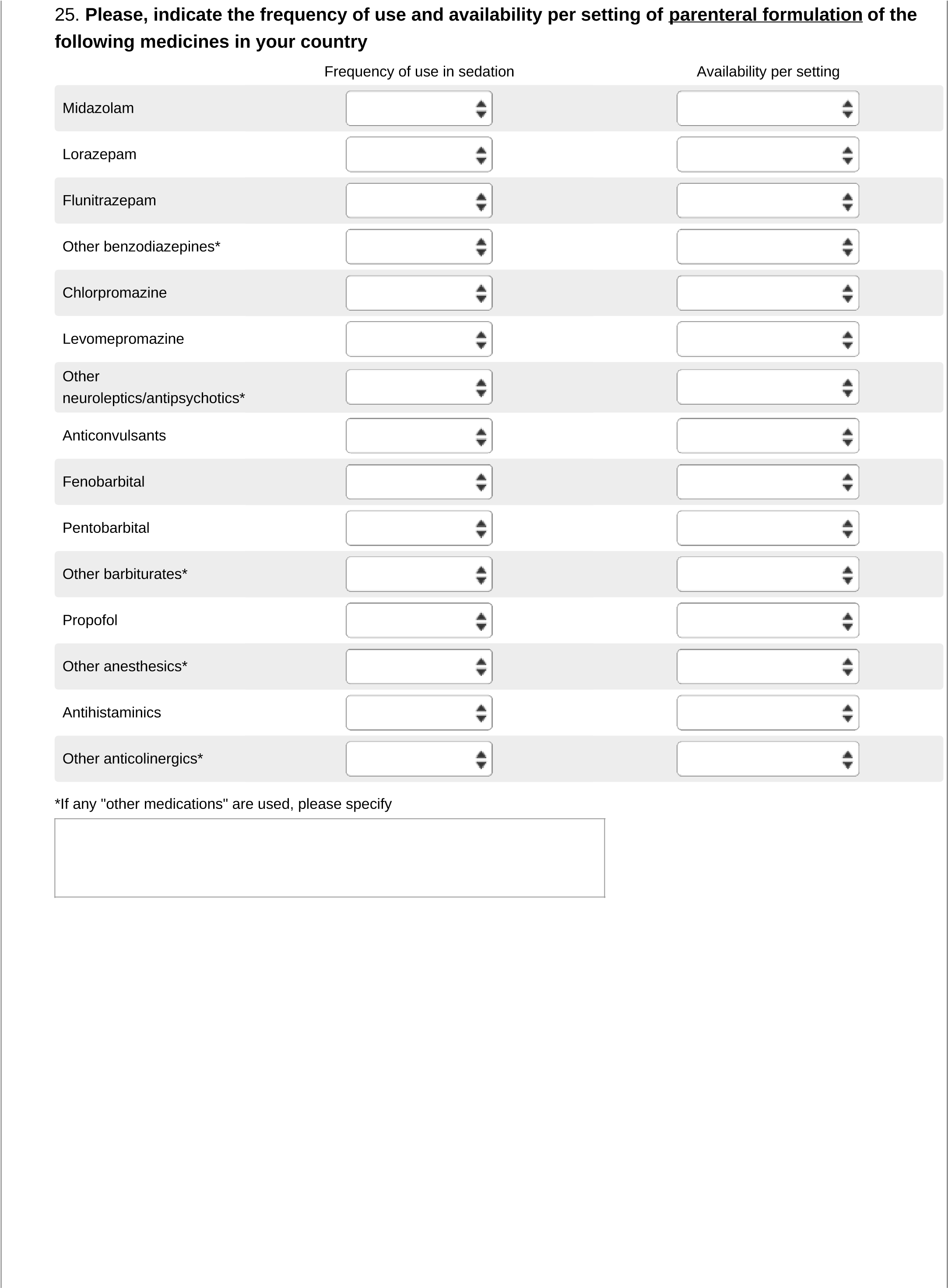


Frequence

Opioid pain medication


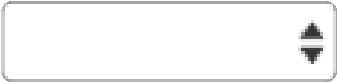


Non-opioid pain

medication


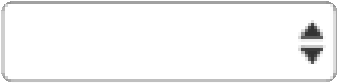


IV hydration


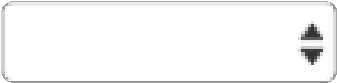


Artificial nutrition


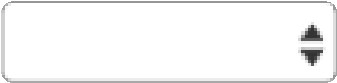


Antibiotic treatment


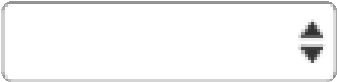


Antithrombotic

medications


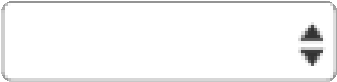


Comments

26

.

**How often are the following co-medications and treatments used with Palliative Sedation?**

.

For these questions we have used the following sources:

-

Arevalo JJ, Rietjens JA, Swart SJ, Perez RSGM, van der Heide A. Day-to-day care in palliative sedation: Survey of nurses’ experiences

with decision-making and performance. Int J Nurs Stud [Internet]. 2013;50(5):613–21

-

Schur S, Masel EK, Mayrhofer M, Watzke HH. 1348Pa Nationwide Survey on Palliative Sedation for Terminally Ill Cancer Patients By

the Austrian Palliative Care (Aupac) Study Group. Ann Oncol [Internet]. 2014;25(suppl_4):iv474-iv474.


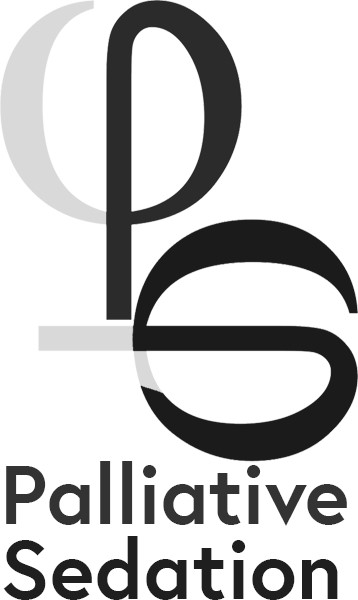


|  |
| --- |
| SECTION 4. Barriers and facilitators to adequate Palliative Sedation in your country |

# Definitions and relevant information

We understand barriers and facilitators as factors that may impede or enhance adequate Palliative Sedation practices.

1. **What are the barriers that hinder Palliative Sedation being carried out with the right medicine?**

Main barrier

Other barriers

1. **What are the facilitators that enable Palliative Sedation to be carried out with the right medicine?**

Main facilitator

Other facilitators

We have developed these questions based on the expertise of the project´s partners.

**Thank you very much for your time!**

**This is the end of the survey**


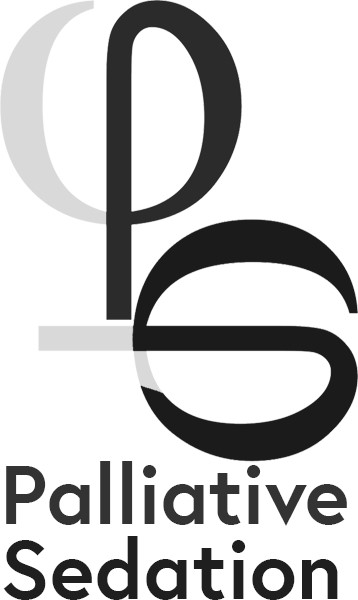


36

.

Please feel free to make any other comments here
